# Evaluation of Compressive Strength of Several Pulp Capping Materials

**DOI:** 10.30476/DENTJODS.2020.83964.1063

**Published:** 2021-03

**Authors:** Ladan Ranjbar Omrani, Zohreh Moradi, Mahdi Abbasi, Mohammad Javad Kharazifard, Seyyedeh Niloufar Tabatabaei

**Affiliations:** 1 Dept. of Restorative Dentistry, School of Dentistry, Tehran University of Medical Sciences, Tehran, Iran; 2 Dental Research Center, Dental Research Institute, Tehran University of Medical Sciences, Tehran, Iran; 3 Postgraduate Student, Dept. of Orthodontics, School of Dentistry, Tehran University of Medical Sciences, Tehran, Iran

**Keywords:** Compressive Strength, Pulp Capping Material, Calcium Hydroxide, Calcium Silicate, Resin-Modified Glass Ionomer

## Abstract

**Statement of the Problem:**

Adequate compressive strength is an important characteristic for an ideal liner.

**Purpose:**

This study aimed to assess the compressive strength of several commonly used liners.

**Materials and Method:**

This in vitro, experimental study evaluated 120 samples fabricated of Dycal, Calcimol LC, Vitrebond, Activa Bioactive, and TheraCal LC (n=24) liners according to the manufacturers’ instructions. The samples were fabricated using a cylindrical stainless steel mold with 6±0.1 mm height and 4±0.1 mm internal diameter. Half of the samples in each group (n=12) underwent compressive strength test immediately after completion of their primary setting while the other half (n=12) underwent compressive strength test after 24 h. During this period, the samples were immersed in deionized water (grade 3) and incubated at 37±1°C and 100% humidity for 24 h. The compressive strength was measured using a universal testing machine. Data were analyzed using two-way ANOVA followed by Tukey’s post-hoc test.

**Results:**

The compressive strength of the five liners was significantly different (p&lt; 0.05). Calcimol LC showed maximum compressive strength both immediately after setting and after 24 h. The compressive strength at 24 h was significantly higher than the primary compressive strength in all groups (p&lt; 0.05).

**Conclusion:**

Within the limitations of this study, it seems that Calcimol LC, Activa Bioactive Liner, and TheraCal LC have adequate compressive strength and can be used alone to provide adequate support for the restorative materials.

## Introduction

Restoration of teeth and preservation of pulp vitality are the main goals of restorative dental treatments [ 1 ]. Pulp exposure may occur due to deep carious lesions or mechanical trauma (such as iatrogenic trauma during tooth preparation), leading to pulp infection and pain. Root canal treatment is relatively invasive, time and cost consuming. Alternatively, vital pulp therapy such as pulp capping may be indicated for such cases [ 2 - 4 ]. Pulp capping treatment is suitable for teeth with no clinical sign/symptom of irreversible pulpitis and necrosis, and no radiographic sign of periapical involvement [ 5 - 6 ]. The success rate of pulp capping treatment ranges from 72.9% to 95.4% [ 7 ]. Pulp capping treatment aims to protect the pulp against physical, chemical, thermal and electrical stimuli (such as galvanic effect of amalgam restorations) as well as microorganisms. It aims to preserve the pulp vitality, seal the dentinal tubules and induce the formation of dentinal bridge by odontoblasts and pulp cells (as the ultimate goal of pulp capping treatment) [ 8 - 10 ]. Pulp capping treatment requires the application of one or more layers of pulp capping agent. Base materials are applied beneath the restorative material when the thickness of the remaining dentin is more than 0.5 mm, and liners are indicated when the thickness of the remaining dentin is less than 0.5mm [ 10 - 12 ]. 

Pulp capping agents are in close contact with the pulp tissue and thus, should be non-toxic and biocompatible. They should be able to provide an optimal seal, minimize microleakage, release fluoride and bond to dentin and restorative materials. They should have low solubility, optimal bio-interactivity and bioactivity, dimensional stability, bactericidal or bacteriostatic property, radiopacity, and adequately high compressive strength [ 11 , 13 - 14 ]. None of the available pulp capping agents have all the above-mentioned properties; thus, their selection depends on the opinion of dental clinician and clinical conditions [ 10 , 15 ]. 

Calcium hydroxide (CH) has long been used as a pulp capping agent due to its excellent antimicrobial property, induction of formation of dentinal bridge, low toxicity and high clinical success rate. It was the gold-standard pulp capping agent for several decades [ 9 , 16 - 17 ]. However, low compressive strength is a major drawback of CH. Thus, it requires the application of glass ionomer (GI) or zinc oxide eugenol to provide adequate compressive strength beneath the restorative materials [ 8 , 18 ]. Calcimol LC is a new CH-based liner with a photo-initiator. It was introduced to the market to eliminate the shortcomings of conventional, auto-polymerizing CH such as Dycal [ 19 ]. 

GIs are also among the commonly used dental materials with advantages such as the ability to absorb and release fluoride and bonding to enamel and dentin. However, moisture sensitivity and low compressive strength are among their drawbacks [ 20 - 21 ]. Some modifications were made in the composition of GIs, which resulted in introduction of resin-modified GIs. Vitrebond is a commercially available resin-modified GI, which is used as a liner in pulp capping treatment of teeth [ 22 ]. Further modifications in the composition of GI powders resulted in advent of GIs containing bioactive glass. Resin matrix was also added to light-cure GIs for further improvement in their mechanical properties, yielding products such as Activa Bioactive base/liner [ 23 - 24 ]. 

Later on, mineral trioxide aggregate (MTA) was introduced to the market with optimal properties such as biocompatibility, induction of dentinal bridge formation, antimicrobial properties, high pH, and radiopacity. Due to drawbacks such as long setting time, and poor mechanical properties such as low compressive strength and difficult handling, Biodentine, was introduced as alternative calcium silicate-based cement to MTA and as a replacement for dentin [ 22 ]. Biodentine is biocompatible, induces dentinal bridge formation, has antimicrobial properties, provides a better sealing than CH, and has a shorter working time and easier handling compared with MTA [ 25 ]. TheraCal LC is a new liner from the family of calcium silicate cements modified with light-cure resin [ 22 ]. 

All the above-mentioned liners should have adequate compressive strength for application under amalgam, composite, metal, or ceramic restorations in order to be able to resist functional and parafunctional stresses in the oral environment. 

Compressive strength test is commonly performed to assess the mechanical properties of restorative materials [ 26 ]. The results of previous studies on the compressive strength of different liners available in dental market are controversial [ 27 - 28 ]. Also, there is a gap of information regarding the compressive strength of cements recently introduced to the market such as TheraCal LC and Calcimol LC. Given that the liners have adequate compressive strength, they can be used alone for pulp capping under the final restorative material without requiring an additional base. Thus, this study aimed to assess the compressive strength of several commonly used liners.

## Materials and Method

This *in vitro*, experimental study measured the compressive strength of 120 samples fabricated from five liners according to ISO 9917-1,2 (2007) for dental cements [ 29 ]. Sample size was calculated to be 24 samples in each of the five cement groups according to a previous study [ 27 ], assuming alpha=0.05, beta=0.2, standard deviation of compressive strength to be 12 MPa, and effect size of 0.375 using one-way ANOVA power analysis feature of PASS 11 software. Of 24 samples in each group, 12 were used for measurement of compressive strength immediately after polymerization while the remaining 12 were used for measurement of compressive strength after 24 h of incubation. Table 1 presents the characteristics of the liners used in this study.

**Table 1:** Characteristics of the liners used in this study

Material	Composition	Manufacturer
Vitrebond (RMGI)	Powder : fluoroaluminosilicate glass	3M ESPE Dental Products, St.
	SiO2, AlF3, ZnO, SrO, cryolite, NH4F, MgO and P2O5.	Paul, MN, USA
	Liquid: polyacrylic acid with pendant methacrylate groups, HEMA (2hydroxyethylmethacrylate), water photo-initiator (camphorquinone (and photosensitizer.	
Activa Bioactive-Base/Liner (RMGI)	Patented bioactive ionic resin	Pulpdent Corporation, Watertown, MA USA
	Patented rubberized resin bioactive glass ionomer with blend of diurethane and other methacrylates with modified polyacrylic acid amorphous silica sodium fluoride	
TheraCal LC	CaO, calcium silicate particles (type III Portland cement), Sr glass, fumed silica, barium sulfate, barium zirconate, and resin containing Bis-GMA, TEGDMA and PEGDMA	Bisco Inc., Schaumburg, IL, USA
Calcimol LC	Urethane dimethacrylate resin, calcium dihydroxide,	VOCO GmbH, Cuxhaven, Germany
	dimethylaminoethyl-methacrylate, and TEGDMA	
Dycal	Base paste: 1,3-butylene glycol disalicylate, zinc oxide, calcium phosphate, calcium tungstate, and iron oxide pigments. Catalyst paste: calcium hydroxide, N-ethyl-o/p-toluene sulfonamide, zinc oxide, titanium oxide, zinc stearate, and iron oxide pigments	Dentsply Tulsa, Dental, Johnson, City, TN, USA

### Fabrication of samples

A cylindrical two-piece stainless steel mold with 6±0.1 mm height and 4±0.1 mm internal diameter was used to fabricate the samples. The internal surface of the mold was uniformly coated with petroleum jelly to enhance removal of the samples after their polymerization. The liners were applied into the molds according to the manufacturers’ instructions and packed. The light-cure liners were applied in 6 increments with 1 mm thickness each, and each layer was light-cured separately for the time period recommended by the manufacturer using a LED 50N light-curing unit (TPC, USA) with a light intensity of 1000 mW/cm^2^. The light intensity of the light-curing unit was periodically checked by a radiometer. Light was irradiated through plexiglass sheets with 1 mm thickness placed on the top and at the bottom of the mold. The study groups were as follows:

### Vitrebond (3M ESPE Dental Products, St. Paul, MN, USA)

The powder and liquid were mixed in 1.4:1 weight ratio (1 scoop of powder and 1 drop of liquid) for 10 to 15 s on a glass slab using a spatula. The mixture was applied into the mold by the spatula in 6 increments each with 1 mm thickness. Each increment was light-cured for 30 s. 

### ACTIVA Bioactive liner (Pulpdent Corporation, Watertown, MA USA)

The base and catalyst pastes were mixed using an auto-mix syringe. To ensure adequate mixing, 1 to 2mm of the mixture was injected and discarded. The mixture was injected into the mold in 6 increments each with 1mm thickness. Each increment was light-cured for 20s. 

### TheraCal LC (Bisco Inc., Schaumburg, IL, USA)

Single paste was injected into the mold in 6 increments each with 1 mm thickness using a special syringe. Each increment was light-cured for 20 s.

### Calcimol LC (VOCO GmbH, Cuxhaven, Germany)

Single paste was injected into the mold in 6 increments each with 1 mm thickness using a special syringe. Each increment was light-cured for 20 s.

### Dycal (Dentsply Tulsa Dental, Johnson City, TN, USA)

The base and catalyst pastes were mixed in equal amounts (1:1) on a pad for 10 s using a spatula to obtain a uniform mixture. The mold was overfilled and then pressure was applied to the surface using a plexiglass slab in order to remove the excess material and prevent the formation of voids. 

After fabrication of samples, their upper and lower surfaces were wet-polished by a 400-grit silicon carbide abrasive paper such that the surface of samples was at the level of the mold and had 90° angle relative to the horizon.

### Compressive strength measurement

Half of the samples in each group (n=12) were used for measurement of compressive strength immediately after the primary setting. For this purpose, the light-polymerized liners were immediately removed from the mold after polymerization. The other surfaces of the samples were also light-cured. The Dycal samples were removed after 3 minutes to allow their auto-polymerization. Next, the samples were inspected for presence of voids, air bobbles or chipping without magnification. Samples with defects were replaced with sound samples. They were then subjected to compressive strength test. In order to assess the compressive strength of the samples after 24 hours, 12 samples in each group were immersed in grade-3 deionized water according to ISO 3696 [ 30 ] and incubated at 37±1°C and 100% humidity for 24 h. Next, they underwent compressive strength test. For the compressive strength test, each sample was vertically placed on the steel surface of universal testing machine. Load was applied along the longitudinal axis of the samples at a crosshead speed of 0.75±0.30 mm/min or 50±16 N/min until fracture. Maximum load at fracture was recorded in Newtons and divided by the diameter of the sample in millimeters to obtain the compressive strength in megapascal (MPa). 

### Statistical analysis

Data were analyzed using SPSS version 25 (SPSS Inc., IL, USA). Two-way ANOVA was applied to assess the effect of time and type of liner on compressive strength. Pairwise comparisons of the groups were carried out using the Tukey’s HSD post-hoc test. *p*≤0.05 was considered statistically significant. 

## Results

Table 2 presents the measures of central dispersion for the compressive strength of the five groups. As shown, Calcimol LC showed maximum compressive strength immediately after setting and also after 24 h. 

**Table 2:** Measures of central dispersion for the compressive strength of the five groups

Liner	Time	Minimum	Maximum	Mean	Std. Deviation
Dycal	0	4.70	12.01	8.13	1.92
	24	14.73	25.33	20.51	2.63
Calcimol LC	0	212.05	406.77	331.38	56.69
	24	165.28	406.12	337.75	71.42
TheraCal LC	0	61.25	100.44	81.47	9.19
	24	87.57	151.45	114.40	22.90
Activa Bioactive liner	0	156.77	324.74	274.96	47.46
	24	245.90	338.70	305.53	25.38
Vitrebond	0	20.11	79.50	55.72	16.42
	24	57.41	76.33	68.61	5.43

Two-way ANOVA showed that the interaction effect of type of liner and time on compressive strength was not significant (*p*= 0.592). However, the effect of time on compressive strength was significant (*p*= 0.003). In other words, the compressive strength of all liners after 24 h was significantly higher than their compressive strength immediately after setting. 

The effect of type of liner on compressive strength was also significant (*p*= 0.000). In other words, the mean compressive strength of the five liners was significantly different. Thus, pairwise comparisons were carried out using the Tukey’s HSD test. 

Table 3 presents the results of this comparison and related p values. The results showed that the compressive strength of Calcimol LC was the highest followed by Activa Bioactive Liner, TheraCal LC, Vitrebond, and Dycal immediately after setting and also after 24 h.

**Table 3:** Pairwise comparisons of the liners regarding their compressive strength

Material 1	Material 2	p Value
Dycal	Calcimol LC	0.000
	TheraCal LC	0.000
	ACTIVA Bioactive liner	0.000
	Vitrebond	0.000
Calcimol LC	Dycal	0.000
	TheraCal LC	0.000
	ACTIVA Bioactive liner	0.000
	Vitrebond	0.000
TheraCal	Dycal	0.000
	Calcimol LC	0.000
	ACTIVA Bioactive liner	0.000
	Vitrebond	0.005
Bioactive liner	Dycal	0.000
	Calcimol LC	0.000
	TheraCal LC	0.000
	Vitrebond	0.000
Vitrebond	Dycal	0.000
	Calcimol LC	0.000
	TheraCal LC	0.005
	ACTIVA Bioactive liner	0.000

## Discussion

This study assessed the compressive strength of five commonly used liners and showed that the compressive strength of the five liners were significantly different. The results showed that the compressive strength of Calcimol LC was the highest followed by Activa Bioactive Liner, TheraCal LC, Vitrebond and Dycal immediately after setting and also after 24 h. The compressive strength at 24 h was significantly higher than the primary compressive strength in all groups. According to ISO 9917 [ 29 ], minimum compressive strength required for pulp capping agents is 50 MPa; however, ideally, these agents should have a compressive strength equal to that of dentin or the permanent restorative material applied over them [ 31 - 32 ]. According to Douglas [ 33 ], compressive strength is the best quality control measure that can be considered by the manufacturers to produce a high-quality restorative material.

Thus, compressive strength is commonly measured as a preliminary test to assess the clinical efficacy of dental materials [ 34 ].

This study assessed the compressive strength of liners immediately after their setting to assess their performance in immediate application of permanent restorative material, which has not been addressed before. The compressive strength of the samples was also measured after 24 h, which is the maximum time often allowed for complete polymerization of liners [ 28 ]. The results showed that Dycal had the lowest compressive strength. Polymerization of Dycal is the result of reaction of CH with 1-methyl tri-methylene di-salicylate, forming amorphous calcium di-salicylate, which is believed to be responsible for low compressive strength of Dycal [ 35 ]. El-Araby *et al*. [ 36 ] showed that replacement of proton with calcium ions during polymerization of Dycal results in chelation of calcium phenolates, which bind to each other with secondary bonds only, causing low mechanical properties of Dycal. Thus, Dycal has high risk of fracture and cannot provide adequate support for the restorative material, if applied alone. Similar to our findings, Natale *et al*. [ 37 ] showed that Dycal had lower compressive strength than other calcium silicate liners such as MTA and Biodentine. 

TheraCal LC is composed of 45% calcium silicate (type III Portland cement) and about 45% resin compounds including UDMA, HEMA, bis-GMA, TEGDMA/tri-EDMA and PEGDMA [ 38 - 39 ], which confer adequate compressive strength to TheraCal LC. 

Stanley *et al*. [ 19 ] compared the compressive strength of Dycal and Prisma visible light-cure Dycal and reported that after 24 h, the visible light-cure Dycal had significantly higher compressive strength than regular Dycal. They attributed this finding to the presence of UDMA light-cure resins in visible light-cure Dycal. Similarly, our study showed that the compressive strength of Calcimol LC, which is a type of light-cure CH, was significantly higher than the compressive strength of Dycal. Calcimol LC had the highest compressive strength in our study. It is mainly composed of resin monomers. UDMA has the highest percentage; butyl hydroxyl toluene and dimethyl amino-ethyl methacrylate have lower percentages. CH accounts for only 2.5% to 5% of this liner [ 40 ]. This resin-rich network is responsible for maximum compressive strength of this liner [ 19 ] superior to that of TheraCal LC. Higher solubility of Dycal also explains its lower compressive strength than Calcimol LC and TheraCal LC [ 19 ]. The same result was reported by Nielsen *et al*. [ 28 ]. They also showed that both TheraCal LC and Dycal had higher compressive strength at 24 h compared with 15 min, which was in line with our findings.

Mitra [ 41 ] showed higher compressive strength of Vitrebond than conventional GI at 1 and 24 h. Also, the compressive strength of Vitrebond at 24 h was higher than that at 1 h. The setting reaction of GI is a gradual process that may take up to 1 month. Eliades *et al*. [ 42 ] showed that the compressive strength of Vitrebond at 1 month was higher than that at 24 h. Increase in compressive strength of GI over time was also noted in our study. Increase in strength of GIs over time can be due to the slow formation of silica matrix during polymerization. Also, it has been demonstrated that strength of GIs depends on gradual degradation of poly-acrylic acid copolymers [ 43 ].

In our study, Activa Bioactive liner ranked second in terms of compressive strength while Yli-Urpo *et al*. [ 27 ] showed that the compressive strength of GIs (conventional and resin-modified types) decreased following the addition of bioactive glass due to their weak bonds to GI matrix during the mixing phase. However, Activa Bioactive liner does not have this separate mixing phase. Activa Bioactive liner has a triple polymerization mechanism including light-cure resin setting, self-cure resin polymerization and self-cure acid-based chemical reactions of GI [ 44 ]. Also, the resin matrix added to it has a double-cure polymerization mechanism, which explains its superior mechanical properties compared with Vitrebond [ 24 ]. Activa Bioactive liner does not contain bisphenol A, bis-GMA or benzoyl peroxide derivatives; however, the exact composition of ionic resin matrix of Activa Bioactive liner has not been disclosed by the manufacturer. Thus, a definite conclusion cannot be drawn regarding the main reason for high compressive strength of this liner. 

This study had an *in vitro* design. Thus, generalization of results to the clinical setting must be done with caution. Clinical studies are required on the compressive strength of these liners. Also, clinical success of a material depends on some other mechanical, physical and chemical factors that need to be addressed in future studies to cast a final judgment regarding an ideal liner for use in the clinical setting. 

## Conclusion

Within the limitations of this study, the results showed that all liners evaluated in this study, except for Dycal, showed adequately high compressive strength according to ISO 9917. Calcimol LC showed maximum compressive strength. 
